# Supplementary material for: Molecular Design, Spectroscopic, DFT, Pharmacological, and Molecular Docking Studies of Novel Ruthenium(III)–Schiff Base Complex: An Inhibitor of Progression in HepG2 Cells
Source: Int J Environ Res Public Health. 2022 Oct 20;19(20):13624. doi: 10.3390/ijerph192013624 (PMC9603487; doi:10.3390/ijerph192013624)
Supplement: Supplementary file 1 [file ijerph-19-13624-s001.zip › ijerph-1878405-supplementary.pdf]

# Supplementary File

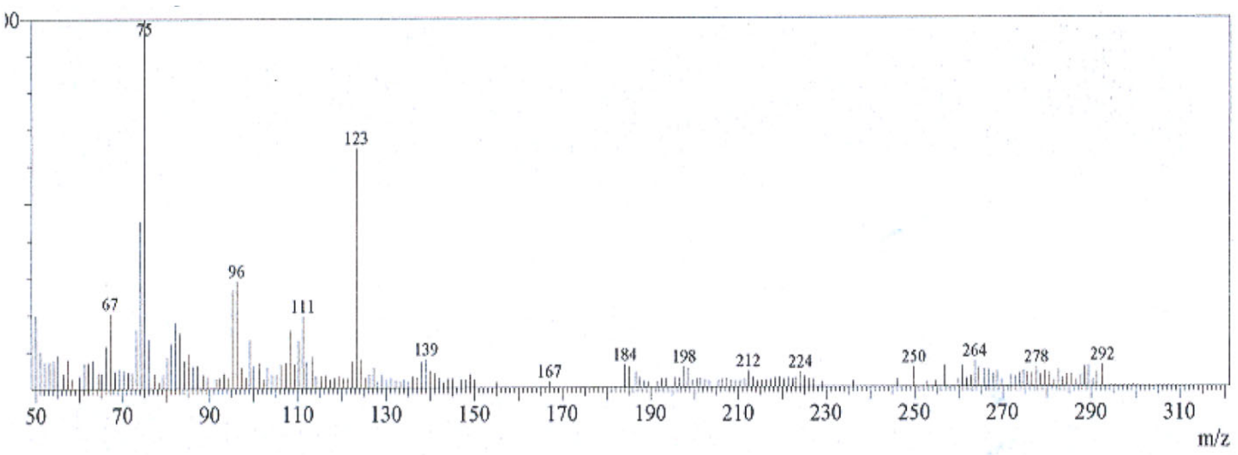


**Figure S1.** The mass spectrum of Schiff base ligand.

---------------------------------------------------------

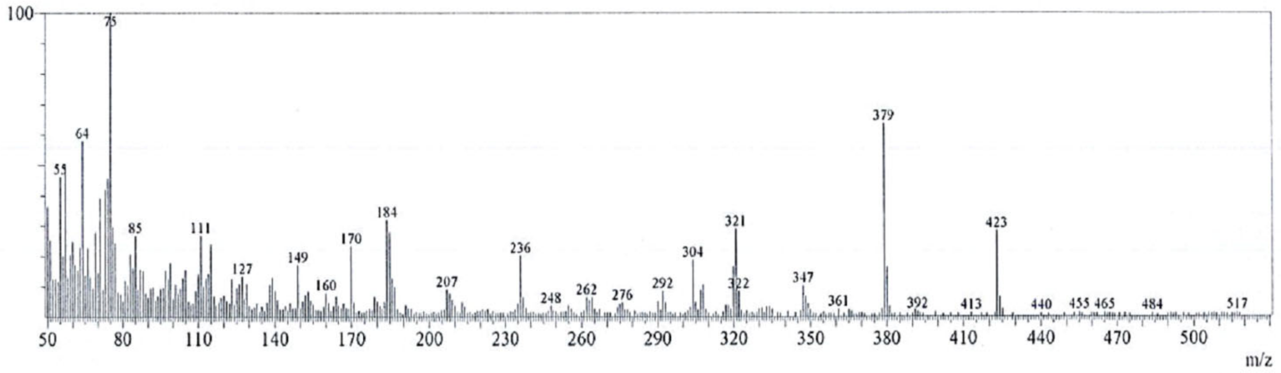


**Figure S2.** The mass spectrum of the ruthenium complex.

------------------------------------------------------------------

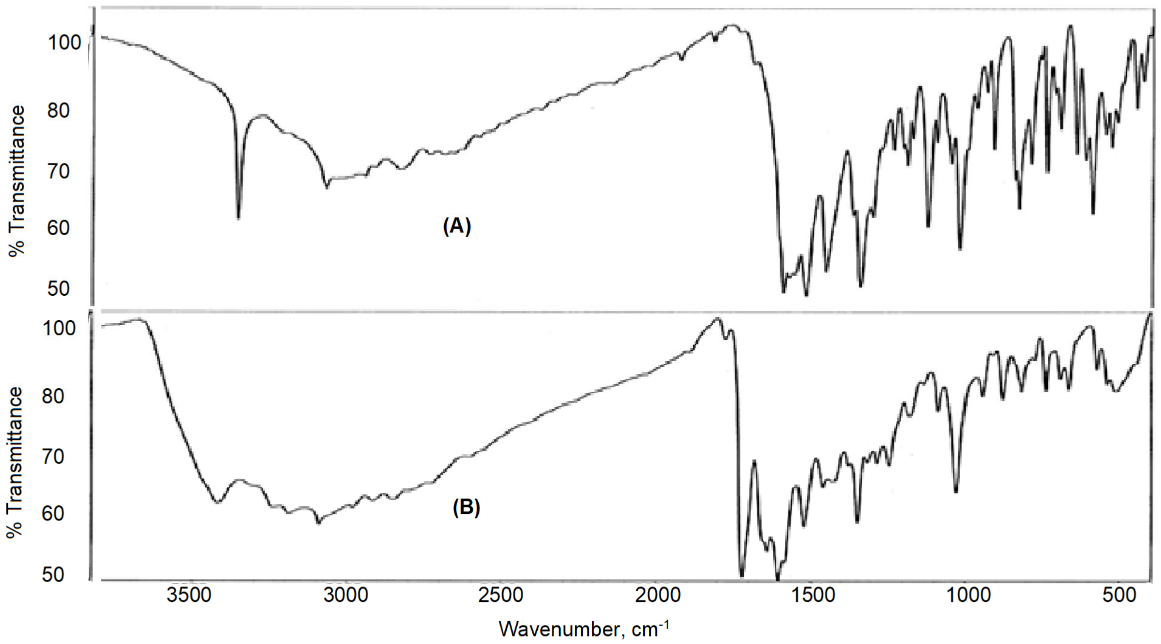


**Figure S3.** The IR spectrum of: **(A)** the Schiff base ligand **L**; **(B)** **RuL** complex.

---------------------------------------------

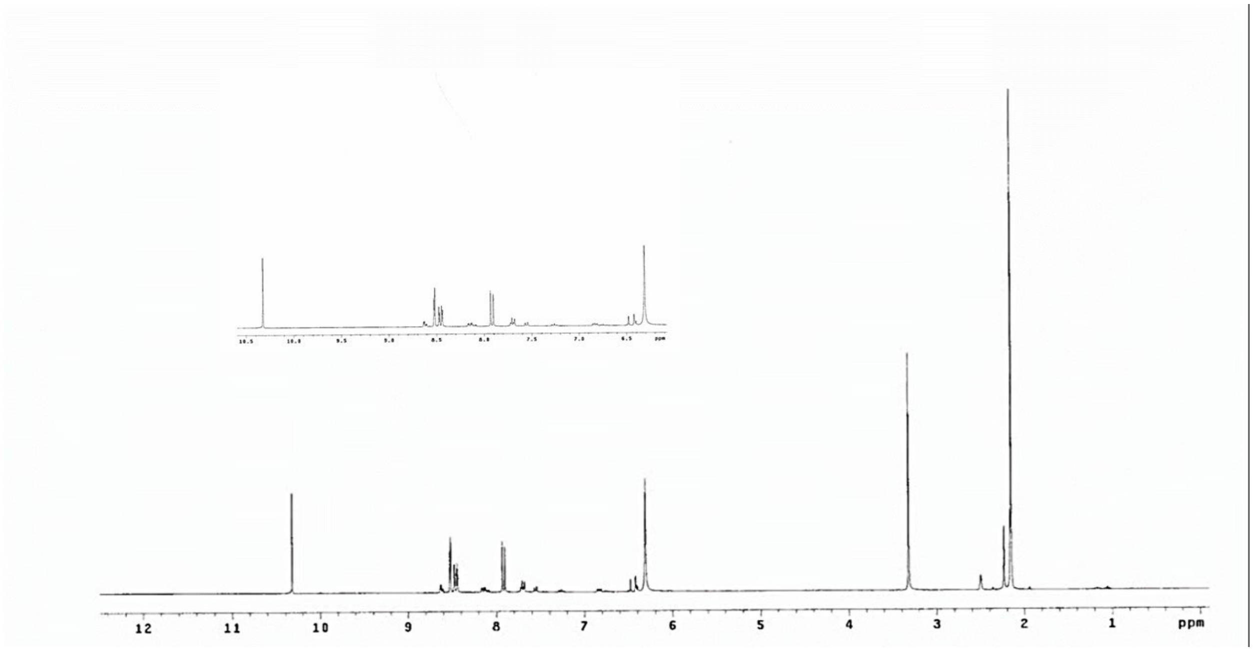


**Figure S4.** The $^{1}H$ NMR spectrum of the Schiff base ligand.

--------------------------------------------------

**Table S1.** The primer sequences of tested genes.

| | Forward | Reverse |
|---|---|---|
| VEGF-A | 5'-TCC TCACAC CAT TGAAAC CA-3' | 5'-GAT CCTGCCCTGTCTCTC TG-3' |
| mTOR | 5’-AGTGGACCAGTGGAAACAGG-3’ | 5’-TTCAGCGATGTCTTGTGAGG-3’ |
| AKT | 5’- TCTATGGCGCTGAGATTGTG -3’ | 5’- CTTAATGTGCCCGTCCTTGT -3’ |
| H2AX | 5′-CGGGCGTCTGTTCTAGTGTT-3′ | 5′-GGTGTAC ACGGCCCACTG-3′ |
| NF KB | 5’- GTGGTGCCTCACTGCTAACT-3’ | 5’- GGATGCACTTCAGCTTCTGT-3’ |
| SND1 | 5’- GGTGGACTACATTAGACCAGCC-3’ | 5’- AGACCTTTGCTGACAAGAGCCTC-3’ |
| CASPASE 3 | 5’- TGGATTATCCTGAGATGGGTTT-3’ | 5’- TTGCTGCATCGACATCTGTA-3’ |
| CASPASE 7 | 5’- ACTGCTCTTGTGCCAAGATG-3’ | 5’- CATGGCTTAAGAGGATGCAG-3’ |
| GAPDH | 5’-ACCCACTCCTCCACCTTTGA-3’ | 5’-CTGTTGCTGTAGCCAAATTCGT-3’ |
